# Nipple reconstruction using spiral-peeling technique during oncoplastic breast-conserving surgery for a patient with small breasts

**DOI:** 10.1007/s00595-024-02892-0

**Published:** 2024-07-05

**Authors:** Yuko Kijima, Munetsugu Hirata, Naotomo Higo, Yumika Nakazawa, Kazuya Shinmura

**Affiliations:** Department of Breast Surgery, School of Medicine, Fujita Health University, 1-98 Dengakugakubo, Kutsukake-cho, Toyoake, Aichi 470-1192 Japan

**Keywords:** Breast cancer, Oncoplastic breast surgery, Core and spiral-cut

## Abstract

Treatment of early breast cancer using breast-conserving surgery (BCS) commonly leads to local control and acceptable cosmetic results. We report a useful technique to achieve symmetry of the breast shape and nipple-areola, with excellent results. A Japanese patient with early breast cancer located in the inner central area of the breast was enrolled in this study. Intraductal spread of breast cancer to the nipple was suspected; however, no invasion was observed outside the nipple wall. We preserved the cylindrical surface, but resected the inner tissue with the top surface of the nipple. After coring the nipple, the remnant cylindrical surface was cut into a spiral shape. Nipple reconstruction using the spiral-peeling technique during oncoplastic breast-conserving surgery (OPBCS) may be useful for patients who desire nipple preservation.

## Introduction

Breast-conserving surgery (BCS) is a well-established treatment for breast cancer that facilitates local disease control with acceptable cosmetic results [1, 2]. In addition to locoregional control and survival, a good cosmetic outcome is one of the main aims of BCT [3, 4]. Resection of the nipple areola with the central area of the breast and simple closure of the defect, both vertically and horizontally, give the breast a particular shape [5].

We previously reported 3 oncoplastic techniques for patients with breast cancer located in the inner area of the breast: 1 involved volume replacement and the other 2 involved displacement [6–8]. We successfully performed oncoplastic breast-conserving surgery (OPBCS) using V-rotation mammoplasty in Japanese patients with small-to-moderately sized breasts. We herein report a modified technique of OPBCS command breast reshaping with immediate reconstruction of the nipple.

## Patient

**Fig. 1:**
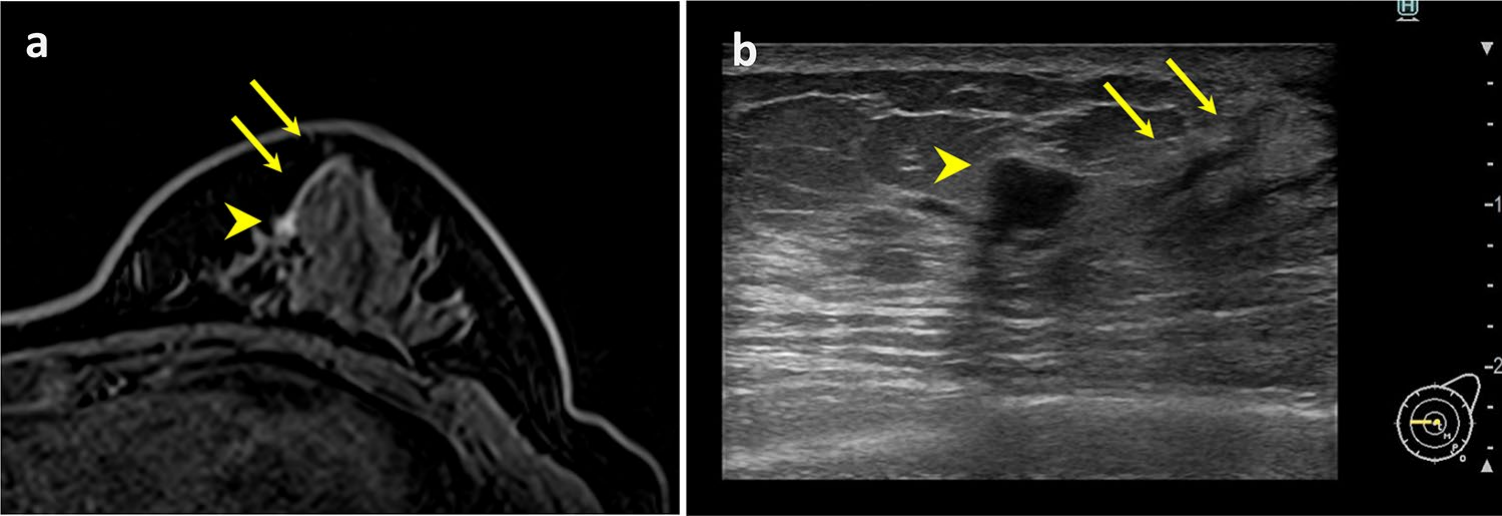
Preoperative MRI and ultrasonography revealed intraductal spread to the nipple. Arrow, intraductal spread to the nipple; arrowhead: cancer lesion. **a** MRI. **b** Ultrasonography

A 44-year-old Japanese patient diagnosed with early-stage breast cancer was enrolled in this study. The lesion was diagnosed as ductal carcinoma in situ and spread toward the nipple. Preoperative MRI and ultrasonography revealed intraductal spread to the nipple (Fig. 1).

She wanted to conserve the breast and did not undergo total mastectomy followed by immediate breast reconstruction. She gave informed consent for V-rotation mammoplasty; the indications for OPBCS using V-rotation mammoplasty were as follows: (1) the cancer lesion was restricted to the inner area of the non-ptotic breast; (2) ductal spread to the nipple-areola was not detected; (3) informed consent was obtained preoperatively after an explanation of the surgical procedure; and (4) she was not worried about the length of the surgical scar. She was also informed of the operative plan and given different surgical options: other oncoplastic surgical techniques, such as a free nipple-areola grafting technique or areola-preserving surgery [9, 10]; and total mastectomy followed by immediate breast reconstruction. Although the intraductal cancerous lesion had spread to the nipple, she refused to undergo mastectomy with breast reconstruction, BCS with nipple resection, BCS with nipple areolar resection such as Grisotti’s method and keyhole resection [11, 12], BCS with nipple-areola resection, or volume replacement using a lattisimus dorsi muscle flap [9]. Thus, we proposed a modification of the V-rotation mammoplasty technique that combined OPBCS, areolar preservation, nipple resection, and nipple reconstruction using the “peeled-nipple” technique. Additional informed consent was obtained regarding the risk of nipple necrosis and the potential for residual cancer.

## Surgical procedure

### Design

**Fig. 2:**
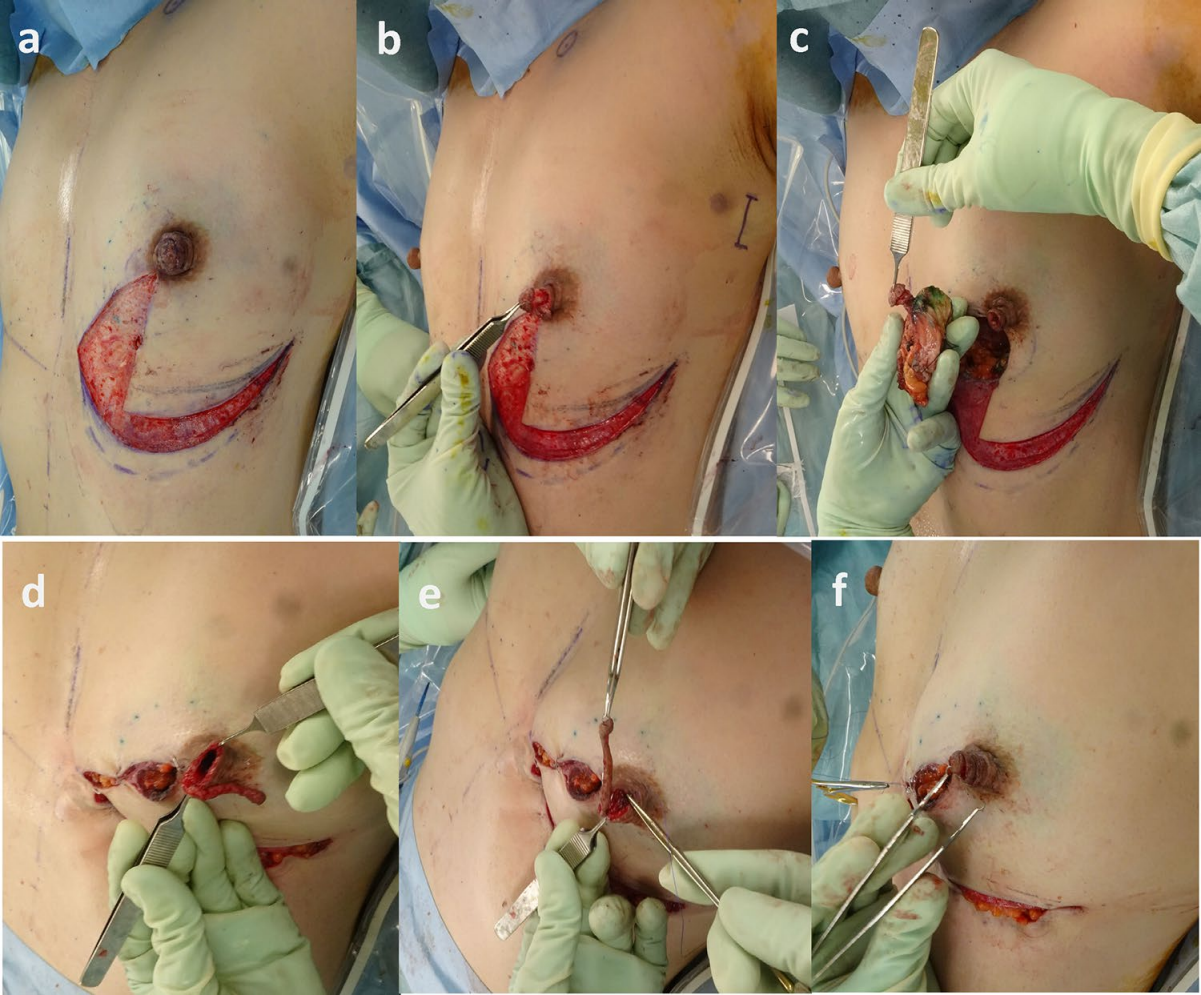
Procedure of OBS using V-mammoplasty. **a** A fan-shaped area in the direction of 8:00 from 7:00 and the crescent area surrounding the inframammary line and its caudal curve were de-epithelialized. **b** A continuous spiral incision was made into the superficial layer of the cylindrical surface of the nipple. We then peeled 3 laps into a continuous spiral. **c** Columnar-shaped breast tissue with the nipple was resected via direct incision at 8:00. **d** A skin-glandular flap in the direction of 6:00–8:00 and the superficial layer of the breast from 4:00 to 6:00 were rotated medially to repair the breast defect. **e** One stitch using an absorbable thread was added to the bottom of the empty space of the nipple to prevent falling of the reconstructed nipple. **e** The cord-formed peeled nipple was piled up in a spiral and sutured

The patient was examined by a breast surgeon (Y.K.) before surgery at the outpatient clinic, and she provided her informed consent. The day before surgery, we designed and created a drawing based on the original technique of V-rotation mammoplasty. The surgical margin was drawn at a 15-mm distance from the cancerous lesion. We then drew 2 straight lines from the edge of the areola in the 7:00 and 8:00 directions, with the patient in the standing position. Two inframammary curves were drawn to determine the area of de-epithelialization (Figs. 2a, 5a).

### Sentinel lymph node biopsy

SNB using the RI and dye method was performed using another incision in the axillary area. One sentinel lymph node was biopsied and histologically examined during the surgery. Metastatic lesions were not observed in the sentinel lymph nodes.

### Oncoplastic breast surgery

**Fig. 3:**
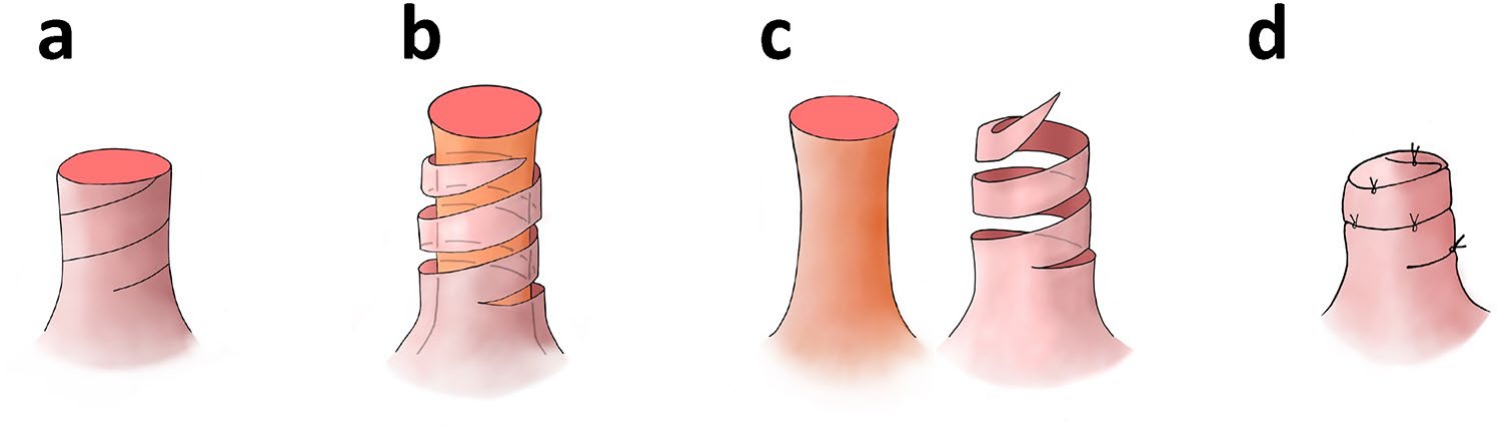
A schematic illustration of nipple reconstruction. **a** A continuous incision line was drawn on the nipple surface using a spiral. **b** A continuous incision was made on the superficial layer of the cylindrical surface of the nipple in a spiral and peeled in a continuous spiral for three laps. **c** The nipple was divided into an internal tissue and a cord-formed nipple surface. **d** The cord-formed nipple was piled up and sutured

**Fig. 4:**
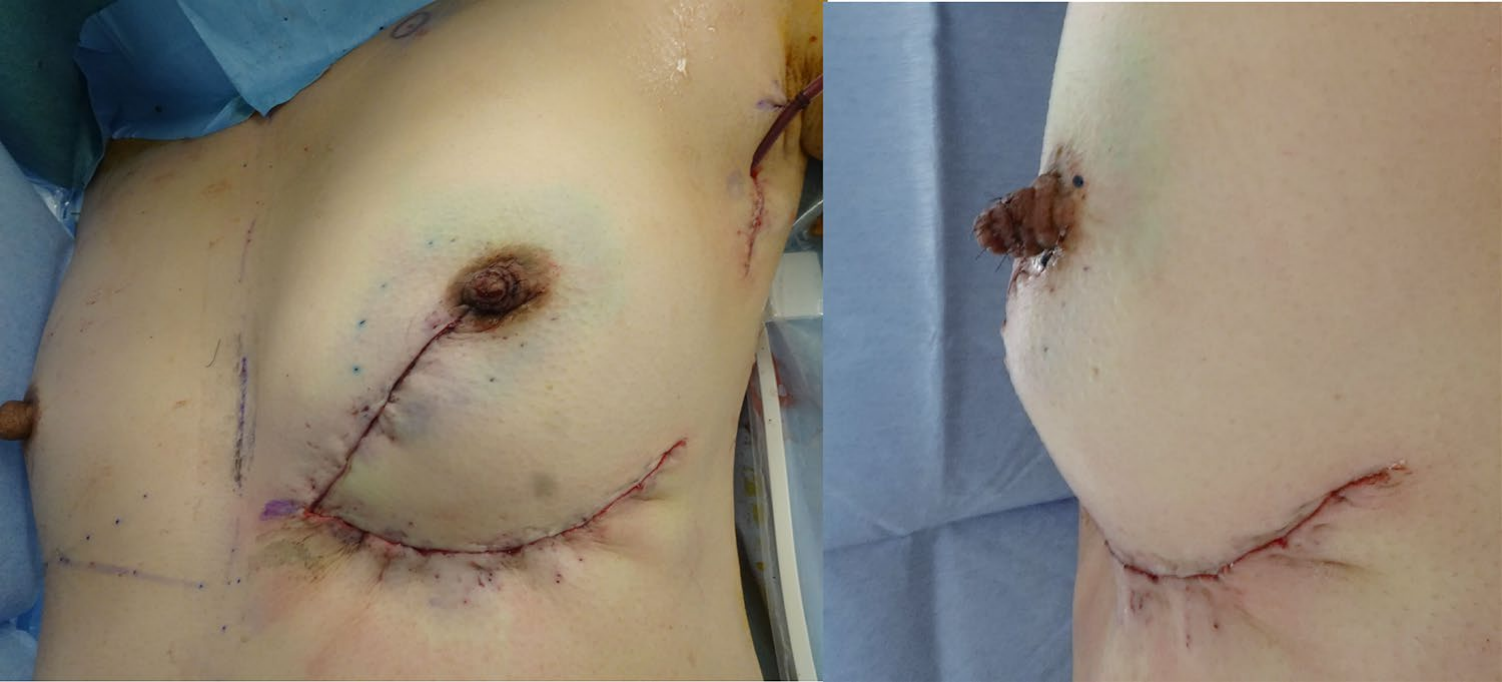
Macroscopic findings at the end of the operation

First, a fan-shaped area in the direction of 8:00 from 7:00, and the crescent area surrounding the inframammary line and its caudal curve were de-epithelialized (Fig. 2a). We made a continuous spiral incision into the superficial layer of the cylindrical surface of the nipple (Figs. 2b, 3a). We then peeled 3 laps in a continuous spiral (Figs. 2b, 3b). The core tissue of the nipple with columnar-shaped breast tissue was resected via direct incision at 8:00 (Figs. 2c, 3c). Four sections of resected breast tissue were examined intraoperatively and were diagnosed as cancer-free. We incised from the internal point in the direction of 8:00 toward the lateral edge along the designed curve. V-shaped skin and subcutaneous tissue were incised along the inframammary line, whereas the skin-breast-fatty tissue was not undermined from the chest wall. All layers in the 6:00–8:00 area and the superficial layer in the 4:00–6:00 area were rotated medially to repair the breast defect (Fig. 1d). One stitch using an absorbable thread was added to the bottom of the empty space of the nipple to prevent falling of the reconstructed nipple (Fig. 1e). The cord-formed peeled nipple was piled in a spiral and sutured using 5-0 nylon (Figs. 2f, 4d). The final findings are shown in Fig. 4.

### Pathological findings

The pathological diagnosis was noninvasive ductal carcinoma of the breast. The nipple was cut into 5 round specimens and examined. No cancer was observed. All margins were cancer-free. The patient underwent irradiation of the remnant breast tissue.

## Results

**Fig. 5:**
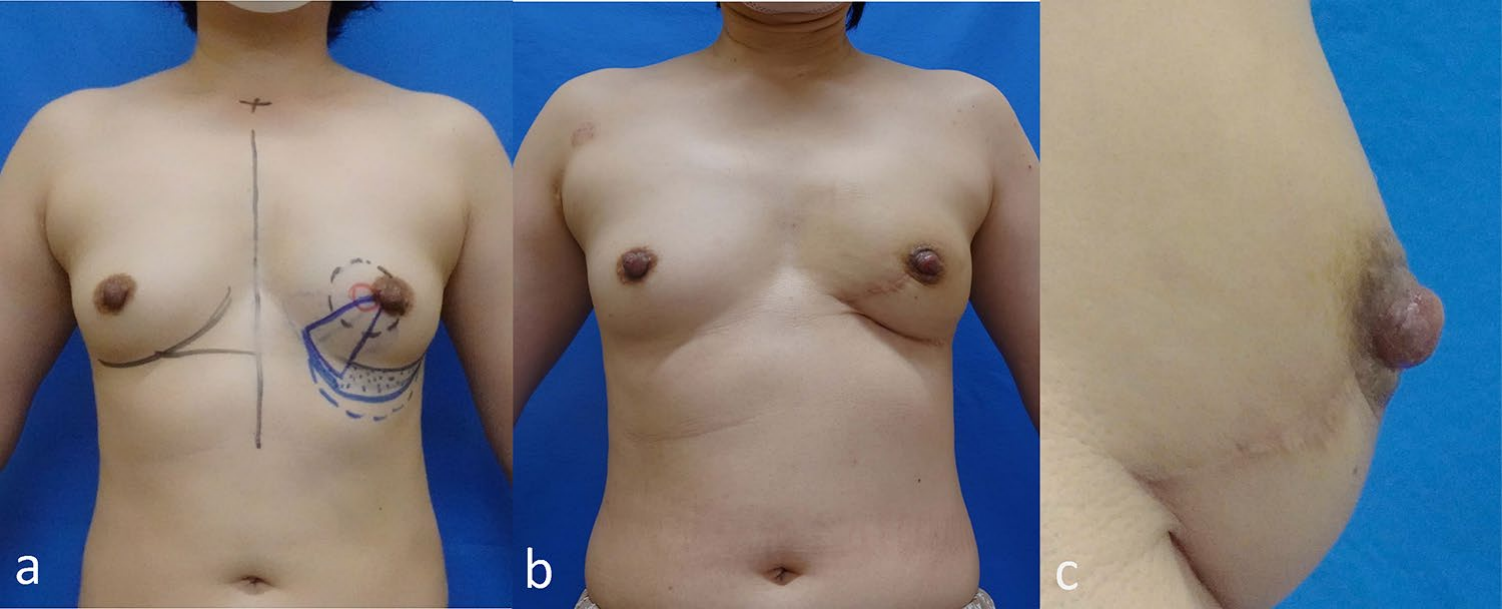
Preoperative (**a**) and 2-year postoperative findings (**b, c**). **a** Preoperative design. **b** The breast shape, size, and position of the NAC were symmetrical at 2-years postoperatively. **c** Good projection of the reconstructed nipple was achieved

There were no postoperative complications (e.g., bleeding, infection, fat necrosis, or blood flow disorder of the reconstructed nipple and breast). The preoperative design and postoperative findings are shown in Fig. 5. Excellent symmetry regarding both the size and shape of the breast was achieved, including the level of the nipple and the inframammary line. Good projection was observed in the left nipple (Fig. 5b, c). The patient did not experience any local or distant recurrence during the 2 years after the operation.

## Discussion

OPBCS has been established rapidly to achieve two major goals in the surgical treatment of breast cancer: oncological safety and excellent cosmetic results [13, 14]. With the introduction of the OPBCS, we have been able to achieve these goals for Japanese patients. For patients with small breasts who lack sufficient volume for displacement, we developed techniques for volume replacement without using muscles [6, 9, 15–17].

We also revealed that OPBCS using the reduction mammoplasty technique was suitable for Japanese patients with large or ptotic breasts, as reported by many researchers in Western countries [12, 18, 19].

BCS for inner and lower lesions is known to result in deformity [20]. We introduced V mammoplasty, which combines elements of volume displacement and replacement for patients with a cancer lesion in the inner area of non-ptotic breasts, as OPBCS [8]. This technique is useful not only for the inner and lower areas but also for the inner, upper, and central areas. In the present case, both oncological safety and good cosmetic results were achieved. Despite involving a small breast and cancerous lesion with a central-inner location, breast reshaping using V-mammoplasty and nipple reconstruction using the peeled cylinder surface of the nipple were successfully performed.

When performing traditional BCS for central breast tumors, NAC and the tumor are excised en bloc, and the defect is closed using wedge excision or purse-string suture. Many women are dissatisfied with their cosmetic results [21, 22]. Carstensea et al. reported the importance of immediate reconstruction of the nipple-areola complex. They recommended three types of reconstruction of NAC during OPBCS [23]. Their techniques yielded excellent results, but both the design and technique may be difficult for breast surgeons. We consider that free nipple-areola grafting can be more easily conducted, and the design and process are simpler [9]; however, its use is limited for patients with large or ptotic breasts.

We reported a straight incision into the skin of the nipple, removed the internal part, and sutured the nipple epidermis [10], in which we selected the OPBCS using the volume replacement technique. From both experiences, we could compare the cosmetic findings of the spiral-peeling technique from a straight incision into the skin of the nipple. As a result, nipples reconstructed using the spiral-peeling technique show superior shape and projection to other nipples.

In this study, the Japanese patient had a small, non-ptotic breast. We planned V-mammoplasty and reconstruction of the nipple by peeling in a continuous spiral for 3 laps using a rolled-up technique. We resected 38 g of tissue; the total operative period was 117 min, and the reconstruction took 63 min. No complications were noted either during or after the surgery. Partial defects of the breast were repaired with skin-fatty tissue in the epigastric area as a V-rotation flap, using basic V-mammoplasty, as outlined in previous reports. This resulted in an adequate volume to fill the defect and make the breast symmetrical. It is important to avoid undermining the skin-breast-fatty tissue from the chest wall as much as possible so that blood flow to the nipple and areolar tissue can be preserved. It remains unclear whether the spiral-peeling technique can be utilized during standard BCS. This is one drawback of the present study. We believe that additional studies are needed to determine the efficacy of this technique for patients hoping for nipple preservation.

One contraindication to this technique is a small or flat nipple. When the outer wall of the nipple is cut and peeled, the intradermal capillary network of the cord form-nipple is the only blood flow that should be preserved. This may be unsuitable for patients presenting with vascular disorders such as diabetes mellitus. Patients with a history of smoking should also be excluded from the indications for nipple reconstruction for the same reason.

## Conclusion

Nipple reconstruction using the spiral-peeled cut technique followed by oncoplastic breast-conserving surgery is useful for patients with breast cancer in the inner to central area of a small non-ptotic breast.
